# The possible role of ribosomal protein S6 kinase 4 in the senescence of endothelial progenitor cells in diabetes mellitus

**DOI:** 10.1186/1475-2840-11-12

**Published:** 2012-02-02

**Authors:** Zhiyong Yin, Linni Fan, Gaosheng Huang, Haichang Wang, Zhe Wang

**Affiliations:** 1 Department of Cardiology, Xijing Hospital, Fourth Military Medical University, Xi'an 710032, China; 2 State Key Laboratory of Cancer Biology and Department of Pathology, Xijing Hospital, Fourth Military Medical University, Xi'an 710032, China

**Keywords:** Diabetes mellitus, Endothelial progenitor cell, RSK4, Senescence

## Abstract

**Background:**

The decrease and dysfunction of endothelial progenitor cells (EPCs) has been assumed as an important cause/consequence of diabetes mellitus (DM) and its complications, in which the senescence of EPCs induced by hyperglycemia may play an immensurable role. However, the mechanisms of EPCs senescence has not been fully investigated. Recently, ribosomal protein S6 kinase 4 (RSK4), a member of serine/threomine (Ser/Thr) kinase family and p53-related gene, is reported to regulate the replicative and stress-induced senescence of different cells.

**Presentation of the hypothesis:**

These above lead to consideration of an evidence-based hypothesis that RSK4 may serve as a mediator of EPCs senescence in DM.

**Testing the hypothesis:**

EPCs of healthy subjects and DM patients are isolated from peripheral blood and incubated with high glucose (HG). Then, the EPCs senescence would be detected by senescence associated β-galactosides (SA-β-gal) staining. Meanwhile, the RSK4 expression is assessed by RT-PCR and western blot. Moreover, overexpressing or RNA interfering of RSK4 in EPCs to investigate the relationship between RSK4 expression and the senescence of EPCs are necessary to substantiate this hypothesis. Also, studies on possible upstream and downstream factors of RSK4 would be explored to reveal the RSK4-mediated senescence pathway in EPCs.

**Implications of the hypothesis:**

If proved, this hypothesis will provide another mediator of EPCs senescence, and may establish a novel pathogenesis for DM and further benefit to the management of DM.

## Background

EPCs are first reported in 1997 [1], which are derived from the bone marrow and could be mobilized to the peripheral circulation in response to stimuli. EPCs have been believed to be angioblasts and contribute to neovascularization, vascular maintenance and repair in adults, and EPCs dysfunction may enhance the risk for cardiovascular disease, DM and tumor [2,3]. Emerging evidence has showed the count and function of EPCs are impaired in DM [4-6]. Moreover, diabetes could alter the subpopulation of EPCs by impairing the production in the bone marrow and decreasing the mobilization from the spleen [7]. Likewise, Jung C et. al found that DM patients had a smaller number of CD34-/CD133+ EPCs, but a larger proportion of apoptotic EPCs [8]. Besides, the reduction of EPCs may augment with an increased number of diseased coronary arteries, which may aggravate the DM and the complications [9]. And it is proved that increased EPCs number could promote the revascularization in asymptomatic type 2 diabetic patients [10,11].

## The mechanisms of EPCs dysfunction in DM

The mechanisms of the EPCs impairment are largely unknown. Reactive oxygen species (ROS) and nitric oxide (NO) are considered as regulators of EPCs [12]. Emerging evidence has found that hyperglycemia, as a type of ROS, could impair vascular endothelial function, and the severity of diabetes is reversely correlated with EPC number and function [13]. However, it is reported that EPCs could tolerate oxidative stress to some extent by upregulating superoxide dismutase (SOD), an enzyme that neutralizes superoxide anion (O_2_-) [14]. Similarly, Hamed S et.al found that EPCs from diabetic patients had higher SOD activity, but lower NO bioavailability than those from the healthy individuals. Nevertheless, when exposed to prolonged hyperglycemia in DM, the function of EPCs are adversely affected by excessive O_2_- generation [15], such as the reendothelialization capacity in vivo [16]. Furthermore, there also exit proofs that optimal glucose control could improve the number and function of EPCs [9,17].

Recently, the senescence of EPCs has been assumed as an important cause/consequence of diabetes and its complications [18], the reasons of which lie in that hyperglycemia in vivo could product free radicals and generate oxidative stress, triggering cellular senescence in DM. However, the mechanisms remain largely unknown.

## RSK4 and senescence

RSK4, ribosomal protein S6 kinase 4, is firstly found as an X-linked gene in patients with mental retardation and most abundantly expressed in brain and kidney [19]. As a member of Ser/Thr kinase family, RSK4 is widely participating in cell signaling pathway by regulating the proliferation and differentiation of cells [20,21]. A large-scale RNAi screen in human cells identifies RSK4 as a new component of the p53 pathway, which could modulate the p53-dependent proliferation arrest on the p21cip1 promoter, either directly or indirectly [22]. Recently, it is reported that RSK4 could regulate replicative and stress-induced senescence [23], and the senescence could be bypassed when RSK4 is inhibited, of which is mediated by p21, but not of p16 or p38MAPKs [24].

## RSK4 and diabetes

As a member of p90^rsk ^family, RSK4 could modulate the synthesis of glucose. Insulin binding to its receptors results in interacting with growth factor receptor-bound protein 2 (Grb2). Grb2 is part of the cascades including RAS, RAF and MEK (MAP2K, Mitogen-activated protein kinase kinase) that leads to activation of mitogen-activated protein kinase (MAPK) and mitogenic responses [25], which includes the activation of glucose synthesis kinase (GSK), resulting in HG. As mentioned above, HG is the main diabetic feature and the cause of EPCs senescence in diabetes. Niehof et. al [26] found that RSK4 might provide a molecular rationale for late-stage complications of kidney and brain in Streptozotocin-induced diabetic rat with hepatic necrotic factor 4α (HNF4α) dysfunction. It is reported that HNF4α could regulate epithelial differentiation and overexpressed HNF4α could cause activation of p21 expression, a senescence mediator, thus inhibiting the cell proliferation [27]. Taken together, it is speculated that RSK4 may mediate EPCs senescence via p21 pathway in DM.

## Presentation of the hypothesis

We assumed that RSK4 might serve as a mediator of EPCs senescence in DM. Hyperglycemia appears to be the most important cause of enhanced EPCs senescence. There are two main pathways involved in senescence: p19/p53 and p16/Rb [28]. p53 or p16 activates p21, which, in turn, can activate retinoblastoma protein (Rb) to shut down the transcription factor (E_2_F) target genes, thus inducing cell growth arrest and senescence [29]. Rosso et.al [30] reported that when cultured under HG, as a kind of ROS, normal EPCs underwent senescent-like growth arrest via the classical p53-dependent senescence pathway. Another study found that p16, together with telomerase, might co-modulate EPCs senescence. Besides, the activation of p38 MAPK pathway also involved in HG-induced EPCs senescence [31]. However, Chen et al. [32] reported that HG enhanced EPC senescence and impaired the migration and tube formation of late EPCs, which were modulated by NO-related rather than oxidative stress-mediated mechanisms through PI3K/Akt/eNOS signaling pathway. Another study [33] also showed PI3K/Akt/eNOS signaling cascade were suppressed in oxidized-LDL and HG treated EPCs, thus leading to the reduced number and the impaired functions of EPC in diabetic patients.

In addition, insulin resistance (IR) may also be a potential factor of EPCs senescence in diabetes. IR could lead to several biochemical alterations, including inflammation and oxidant stress, which leads to the dysfunction of EPCs via the following two pathways: PI3K-PDK1-Akt and RAS-MAPK-p38 pathway [34-36]. However, it is not clear that whether this dysfunction of EPCs is senescence-related and what the senescence mediators are.

Based above, HG or IR could trigger signaling pathways, leading to the senescence of EPCs in DM. For now, PI3K-Akt-eNOS and p53-dependent pathway are considered to be linked to EPCs senescence [37].

**Figure 1:**
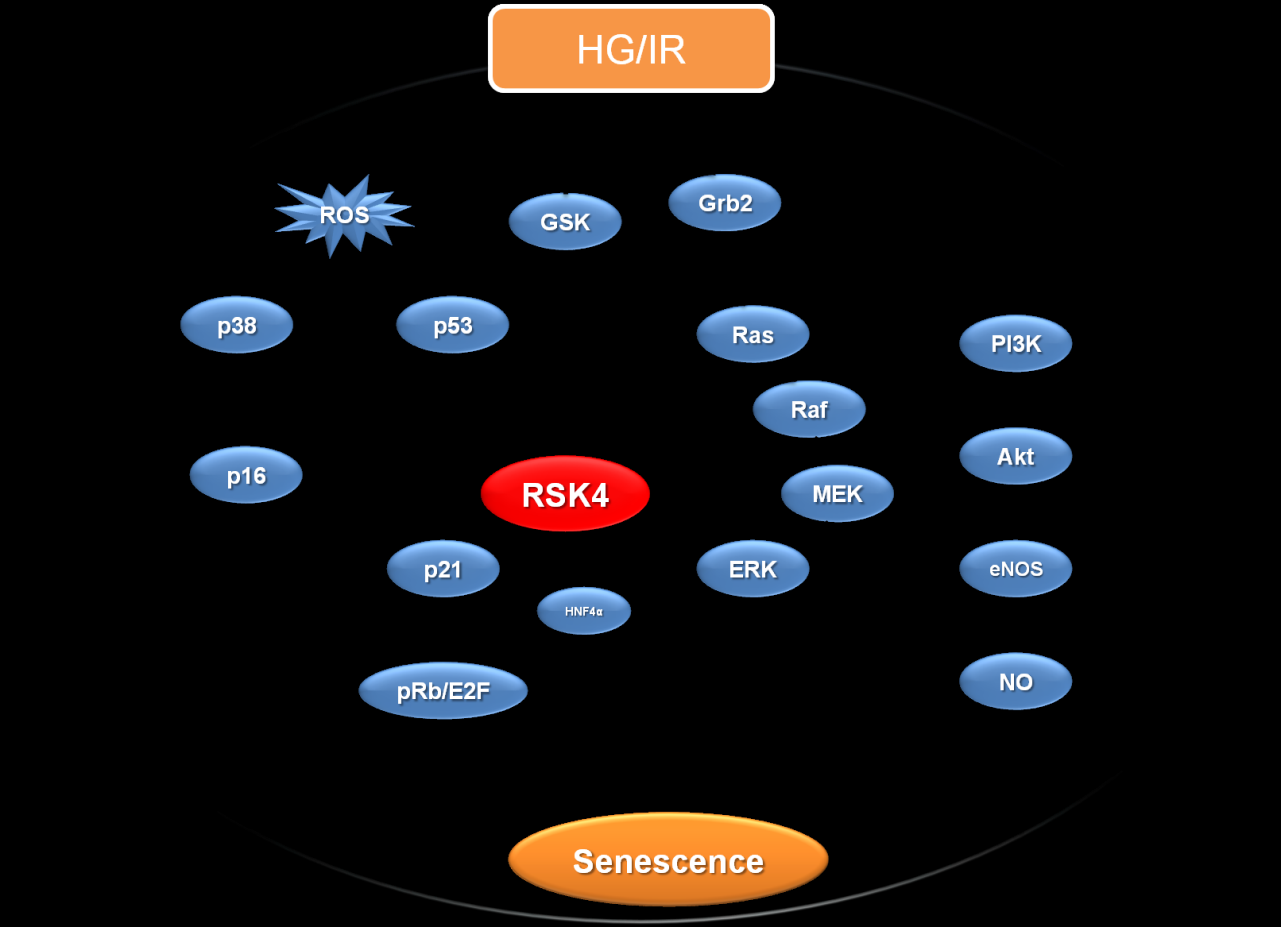
**The possible signaling pathways of EPCs senescence**. In diabetes, HG and/or IR might induce EPCs senesecne via the following pathways. HG and/or IR could inhibit the PI3K-Akt-eNOS pathway, resulting in the decrease of NO, which might induce the EPCs senescence. At the same time, HG and/or IR could be a kind of ROS and induce senescence through the classical p16 and p53 dependent senescence pathway, in which p38 is also invovled. Moreover, we conjecture that HG and/or IR could activate the insulin receptor mediated Ras-MEK-RSK4 pathway, resulting in on one hand the EPCs senescence mediated by RSK4 via p21 signaling pathway and a more production of glucose on the other hand. In additon, RSK4 could be a cadidate gene for HNF4α, which activates p21 and thus inhibit the cell proliferation in diabetes. HG: high glucose; IR: insulin resistance; ROS: reactive oxygen species; GSK: glucose synthesis kinase; HNF4α: hepatic necrotic factor 4α.

Given that RSK4, a p53-related gene, participates in Ras-MEK-ERK pathway and could regulate senescence, we postulate that RSK4 might be a mediator in EPCs senescence in DM. If true, it will provide more information about the pathogenesis of diabetes and new therapeutic targets for diabetic patients. The possible signaling pathway of EPCs senescence is listed as Figure 1.

## Testing the hypothesis

Our hypothesis demonstrates RSK4 protein might take a part in the senescence of EPCs. To testify the hypothesis, EPCs of healthy subjects and DM patients are isolated from peripheral blood and incubated with high glucose. Then, the EPCs senescence would be detected by SA-β-gal staining, and there might present an elevated number of SA-β-gal-positive EPCs. Meanwhile, the RSK4 expression is assessed by RT-PCR and western blot to find out whether it can be upregulated, which could provide an effective evidence for the hypothesis. Moreover, overexpressing or RNA interfering of RSK4 in EPCs to investigate the relationship between RSK4 expression and the senescence of EPCs are necessary to substantiate this hypothesis. Also, studies on possible upstream and downstream factors of RSK4 would be explored to reveal the RSK4-mediated senescence pathway in EPCs.

## Implications of the hypothesis

As above, our new hypothesis might be another explanation to the EPCs senescence in DM. These findings may provide insight into a novel pathophysiological mechanism of DM and may offer new therapeutic opportunities in the future.

## Abbreviations

Akt, PKB: protein kinase B; DM: Diabetes mellitus; E2F: Transcription factor; eNOS: Endothelial nitric oxide synthase; EPCs: Endothelial progenitor cells; ERK: Extracellular regulated protein kinases; Grb2: Growth factor receptor-bound protein 2; GSK: Glucose synthesis kinase; HG: High glucose; HNF4α: Hepatic necrotic factor 4α; IR: Insulin resistance; LDL: Low density lipoprotein; MAPK: Mitogen-activated protein kinase; MEK, MAP2K: mitogen-activated protein kinase kinase; NO: Nitric oxide; PDPK1: 3-phosphoinositide-dependent protein kinase; PI3K: Phosphatidylinositol 3-kinase; Rb: Retinoblastoma protein; ROS: Reactive oxygen species; RSK4: Ribosomal protein S6 kinase 4; SA-β-gal: Senescence associated β-galactosides; Ser/Thr: Serine/threomine; SOD: Superoxide dismutase.

## Competing interests

The authors declare that they have no competing interests.

## Authors' contributions

WH and WZ conceived the hypothesis. All authors contributed to the manuscript, and revisions were carried out by HG. All authors have read and approved the final manuscript.
